# 5,6-Dioxo-1,10-phenanthrolin-1-ium nitrate

**DOI:** 10.1107/S1600536807066172

**Published:** 2007-12-18

**Authors:** Alireza Abbasi, Alireza Badiei, Sara Tarighi

**Affiliations:** a School of Chemistry, University College of Science, University of Tehran, Tehran, Iran

## Abstract

In the title salt, C_12_H_7_N_2_O_2_^+^·NO_3_^−^, the monoprotonated cation is connected to the nitrate anion by a hydrogen bond. Weak C—H⋯O hydrogen bonds hold the planar cations together in a layer structure.

## Related literature

For related literature, see Fujihara *et al.* (2004 ▶); Larsson & Ohström (2004 ▶). For the bromide salt, see: Bomfim *et al.* (2003 ▶).
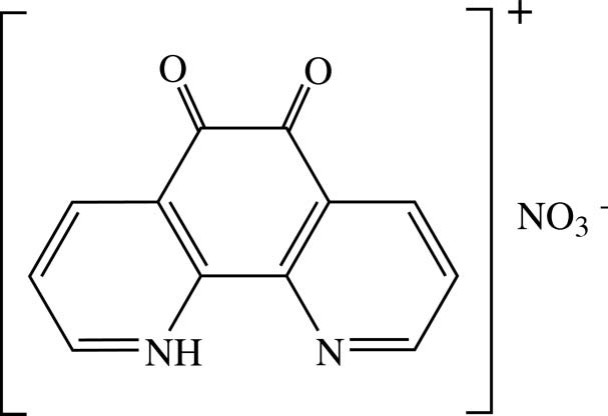


## Experimental

### 

#### Crystal data

C_12_H_7_N_2_O_2_^+^·NO_3_^−^
*M* *_r_* = 273.21
Monoclinic, 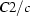

*a* = 14.4860 (19) Å
*b* = 12.5177 (13) Å
*c* = 13.4535 (16) Å
β = 101.720 (12)°
*V* = 2388.7 (5) Å^3^
*Z* = 8
Mo *K*α radiation
μ = 0.12 mm^−1^
*T* = 290 (2) K
0.30 × 0.10 × 0.08 mm

#### Data collection

Oxford Diffraction Xcalibur 3 CCD diffractometer
Absorption correction: numerical (*X-RED*; Stoe & Cie, 1997 ▶) *T*_min_ = 0.960, *T*_max_ = 0.989
7311 measured reflections
2095 independent reflections
635 reflections with *I* > 2σ(*I*)
*R*_int_ = 0.068

#### Refinement

*R*[*F* ^2^ > 2σ(*F* ^2^)] = 0.038
*wR*(*F* ^2^) = 0.060
*S* = 0.83
2095 reflections
182 parameters
H-atom parameters constrained
Δρ_max_ = 0.31 e Å^−3^
Δρ_min_ = −0.14 e Å^−3^

### 

Data collection: *CrysAlis CCD* (Oxford Diffraction, 2003 ▶); cell refinement: *CrysAlis CCD*; data reduction: *CrysAlis RED* (Oxford Diffraction, 2003 ▶); program(s) used to solve structure: *SHELXS97* (Sheldrick, 1997 ▶); program(s) used to refine structure: *SHELXL97* (Sheldrick, 1997 ▶); molecular graphics: *DIAMOND* (Brandenburg, 2001 ▶); software used to prepare material for publication: *PLATON* (Spek, 2003 ▶).

## Supplementary Material

Crystal structure: contains datablocks I, global. DOI: 10.1107/S1600536807066172/ng2405sup1.cif

Structure factors: contains datablocks I. DOI: 10.1107/S1600536807066172/ng2405Isup2.hkl

Additional supplementary materials:  crystallographic information; 3D view; checkCIF report

## Figures and Tables

**Table 1:** Hydrogen-bond geometry (Å, °)

*D*—H⋯*A*	*D*—H	H⋯*A*	*D*⋯*A*	*D*—H⋯*A*
N1—H1⋯O5	0.86	1.95	2.703 (4)	145
C8—H8⋯O5^i^	0.93	2.48	3.396 (4)	167
C9—H9⋯O4^i^	0.93	2.71	3.359 (5)	128
C4—H4⋯O4^ii^	0.93	2.61	3.323 (4)	134
C3—H3⋯O3^ii^	0.93	2.40	3.209 (4)	146
C10—H10⋯O1^iii^	0.93	2.47	3.318 (5)	151
C5—H5⋯O2^iv^	0.93	2.37	3.266 (5)	162

Symmetry codes: (i) 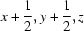; (ii) 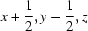; (iii) 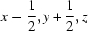; (iv) 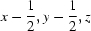.

**Fig. 1.**
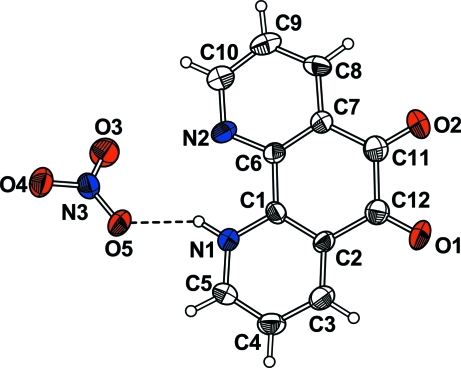
Molecular structure of (I), with 50% probability displacement ellipsoids. H atoms are shown as circles of arbitrary radii.

**Fig. 2.**
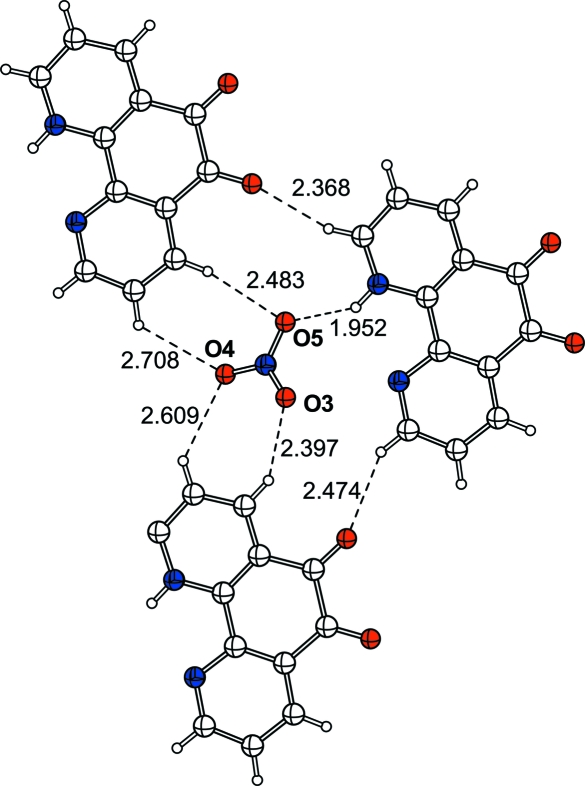
Packing view for (I), showing the hydrogen bonds as dashed lines.

## supplementary crystallographic information

### Comment

From a survey of the Cambridge Structural Database [version 5.28, updated Jan. 2007], we found two complexes containing O-protonated pdon (Fujihara, *et al.*, 2004; Larsson, & Ohström, 2004). However, up to now, only one molecule is reported for the N-protonated pdon (C_12_H_7_N_2_O_2_^+^, Hpdon) with bromide ions as counter ion (Bomfim *et al.*, 2003).

The molecular structure and atom-labeling scheme for (I) are shown in Fig. 1. There is a relatively strong hydrogen bond between the nitrate ion and to the protonated nitrogen atom in Hpdon (N1—H1···O5, 2.703 (4) Å). Weak C–H···O hydrogen bonds hold the approximately planar cations together in a layer structure (Fig. 2). The packing of the layers is similar to that in the bromide salt of Hpdon (Bomfim *et al.*, 2003) without specific directional interactions between the layers. Ring A (C1–C5/N1), B (C6–C10/N2) and C (C1/C2/C12/C11/C7/C6) in the cation (C_12_H_7_N_2_O_2_^+^, Hpdon) is approximately planar. The interplanar angle between the least-square planes defined by ring A and two other rings, B and C, are 1.69 (5)° and 1.17 (5)°, respectively. While, rings B and C are almost planar, 0.52 (5)°. However, the C—N—C angle is affected by protonation and causes to increase from 116.6 (3)° to 122.7 (3)° in the rings B and A, respectively. Similar effect has also been shown in the previously reported Hpdon bromide by increasing from 116.8 (3)° to 123.2 (3)° for the corresponding values. The comparison of the torsion angle, O1—C12—C11—O2, 2.5 (6)°, in (I) with the molecular pdon indicates that the N-protonation in (I) doesn't significantly affect on dione groups.

### Experimental

The title compound was obtained during the reaction of thorium nitrate, Th(NO_3_)_4_.5H_2_O (Merck, 99%), and 1,10-phenanthroline-5,6-dione (Aldrich, 97%) in ethanolic solution. Pale-yellow crystals of Hpdon were taken from the obtained greenish precipitates. The presence of water in the ethanolic solution and the high basicity of dione ligand can be the reason for the protonation, providing the C_12_H_7_N_2_O_2_^+^, Hpdon cation. Thin fragile layer crystals were obtained by recrystallization from acetonitrile.

### Refinement

All H atoms were geometrically positioned and constrained to ride on their parent atoms, with *U*_iso_(H) = 1.2 times *U*_eq_(C,*N*).

### Crystal data

**Table:**

C_12_H_7_N_2_O_2_^+^·NO_3_^–^	*F*_000_ = 1120
*M**_r_* = 273.21	*D*_x_ = 1.519 Mg m^−^^3^
Monoclinic, *C*2/*c*	Mo *K*α radiation λ = 0.71073 Å
Hall symbol: -C 2yc	Cell parameters from 7311 reflections
*a* = 14.4860 (19) Å	θ = 3.5–25.0º
*b* = 12.5177 (13) Å	µ = 0.12 mm^−^^1^
*c* = 13.4535 (16) Å	*T* = 290 (2) K
β = 101.720 (12)º	Needle, pale-yellow
*V* = 2388.7 (5) Å^3^	0.30 × 0.10 × 0.08 mm
*Z* = 8	

### Data collection

**Table:**

Oxford Diffraction Xcalibur 3 CCD diffractometer	2095 independent reflections
Radiation source: fine-focus sealed tube	635 reflections with *I* > 2σ(*I*)
Monochromator: graphite	*R*_int_ = 0.068
Detector resolution: 12 pixels mm^-1^	θ_max_ = 25.0º
*T* = 290(2) K	θ_min_ = 3.8º
ω–scans at different φ	*h* = −17→17
Absorption correction: numerical(X-RED; Stoe & Cie, 1997)	*k* = −14→14
*T*_min_ = 0.960, *T*_max_ = 0.989	*l* = −10→15
7311 measured reflections	

### Refinement

**Table:**

Refinement on *F*^2^	Hydrogen site location: inferred from neighbouring sites
Least-squares matrix: full	H-atom parameters constrained
*R*[*F*^2^ > 2σ(*F*^2^)] = 0.038	*w* = 1/[σ^2^(*F*_o_^2^) + (0.01*P*)^2^] where *P* = (*F*_o_^2^ + 2*F*_c_^2^)/3
*wR*(*F*^2^) = 0.060	(Δ/σ)_max_ < 0.001
*S* = 0.83	Δρ_max_ = 0.31 e Å^−^^3^
2095 reflections	Δρ_min_ = −0.14 e Å^−^^3^
182 parameters	Extinction correction: SHELXL97 (Sheldrick, 1997), Fc^*^=kFc[1+0.001xFc^2^λ^3^/sin(2θ)]^-1/4^
Primary atom site location: structure-invariant direct methods	Extinction coefficient: 0.00072 (4)
Secondary atom site location: difference Fourier map	

### Special details

**Table:**

Geometry. All e.s.d.'s (except the e.s.d. in the dihedral angle between two l.s. planes) are estimated using the full covariance matrix. The cell e.s.d.'s are taken into account individually in the estimation of e.s.d.'s in distances, angles and torsion angles; correlations between e.s.d.'s in cell parameters are only used when they are defined by crystal symmetry. An approximate (isotropic) treatment of cell e.s.d.'s is used for estimating e.s.d.'s involving l.s. planes.
Refinement. Refinement of *F*^2^ against ALL reflections. The weighted *R*-factor *wR* and goodness of fit *S* are based on *F*^2^, conventional *R*-factors *R* are based on *F*, with *F* set to zero for negative *F*^2^. The threshold expression of *F*^2^ > σ(*F*^2^) is used only for calculating *R*-factors(gt) *etc*. and is not relevant to the choice of reflections for refinement. *R*-factors based on *F*^2^ are statistically about twice as large as those based on *F*, and *R*- factors based on ALL data will be even larger.

### Fractional atomic coordinates and isotropic or equivalent isotropic displacement parameters (Å^2^)

**Table:**

	*x*	*y*	*z*	*U*_iso_*/*U*_eq_	
O1	0.85417 (17)	−0.13522 (19)	0.12439 (18)	0.0673 (8)	
O2	0.85003 (19)	0.08376 (19)	0.1238 (2)	0.0834 (10)	
O3	0.30299 (14)	0.0377 (2)	0.07097 (16)	0.0649 (7)	
O4	0.22236 (15)	−0.03456 (19)	0.17219 (15)	0.0613 (6)	
O5	0.34903 (16)	−0.11186 (18)	0.14632 (16)	0.0622 (7)	
N1	0.52797 (19)	−0.1426 (2)	0.12265 (18)	0.0439 (9)	
H1	0.4776	−0.1063	0.1230	0.053*	
N2	0.5205 (2)	0.0692 (2)	0.1274 (2)	0.0488 (9)	
N3	0.29121 (19)	−0.0361 (3)	0.1292 (2)	0.0476 (8)	
C1	0.6089 (3)	−0.0896 (3)	0.1224 (2)	0.0370 (9)	
C2	0.6903 (3)	−0.1473 (3)	0.1211 (2)	0.0374 (10)	
C3	0.6870 (3)	−0.2573 (3)	0.1201 (2)	0.0528 (11)	
H3	0.7411	−0.2969	0.1191	0.063*	
C4	0.6018 (3)	−0.3086 (3)	0.1207 (2)	0.0550 (11)	
H4	0.5986	−0.3828	0.1198	0.066*	
C5	0.5228 (3)	−0.2490 (3)	0.1224 (2)	0.0502 (11)	
H5	0.4657	−0.2826	0.1235	0.060*	
C6	0.6062 (3)	0.0275 (3)	0.1258 (2)	0.0380 (9)	
C7	0.6867 (3)	0.0879 (3)	0.1278 (2)	0.0421 (10)	
C8	0.6798 (3)	0.1978 (3)	0.1312 (2)	0.0534 (13)	
H8	0.7320	0.2411	0.1319	0.064*	
C9	0.5922 (3)	0.2415 (3)	0.1336 (2)	0.0589 (12)	
H9	0.5852	0.3151	0.1369	0.071*	
C10	0.5151 (3)	0.1749 (3)	0.1308 (3)	0.0589 (13)	
H10	0.4568	0.2058	0.1314	0.071*	
C11	0.7780 (3)	0.0351 (4)	0.1244 (2)	0.0526 (10)	
C12	0.7807 (3)	−0.0894 (3)	0.1227 (2)	0.0469 (11)	

### Atomic displacement parameters (Å^2^)

**Table:**

	*U*^11^	*U*^22^	*U*^33^	*U*^12^	*U*^13^	*U*^23^
O1	0.0405 (19)	0.064 (2)	0.102 (2)	0.0165 (15)	0.0270 (16)	0.0046 (15)
O2	0.051 (2)	0.062 (2)	0.146 (2)	−0.0150 (16)	0.0412 (18)	−0.0002 (17)
O3	0.0736 (19)	0.0573 (18)	0.0696 (16)	0.0079 (15)	0.0282 (13)	0.0205 (15)
O4	0.0508 (18)	0.0664 (15)	0.0730 (16)	0.0081 (16)	0.0276 (13)	0.0048 (14)
O5	0.0453 (17)	0.0477 (18)	0.0977 (18)	0.0163 (14)	0.0242 (14)	0.0116 (15)
N1	0.036 (2)	0.038 (2)	0.0605 (19)	0.0038 (18)	0.0143 (17)	0.0013 (19)
N2	0.054 (2)	0.035 (2)	0.0620 (19)	0.0086 (17)	0.0214 (18)	−0.0027 (17)
N3	0.041 (2)	0.051 (2)	0.053 (2)	−0.005 (2)	0.0144 (17)	−0.001 (2)
C1	0.034 (3)	0.038 (3)	0.041 (2)	−0.007 (2)	0.012 (2)	−0.003 (2)
C2	0.037 (3)	0.033 (3)	0.044 (2)	0.008 (2)	0.011 (2)	0.006 (2)
C3	0.056 (3)	0.043 (3)	0.065 (3)	0.009 (3)	0.023 (2)	0.002 (2)
C4	0.067 (4)	0.034 (3)	0.066 (3)	0.001 (3)	0.020 (2)	0.004 (2)
C5	0.050 (3)	0.035 (3)	0.068 (3)	−0.007 (2)	0.017 (2)	0.000 (2)
C6	0.037 (3)	0.035 (3)	0.042 (2)	−0.001 (3)	0.0093 (19)	−0.010 (3)
C7	0.038 (3)	0.036 (3)	0.053 (2)	0.002 (2)	0.011 (2)	0.002 (2)
C8	0.060 (4)	0.030 (3)	0.070 (3)	−0.005 (2)	0.012 (2)	0.003 (2)
C9	0.073 (4)	0.037 (3)	0.065 (3)	0.015 (3)	0.010 (3)	−0.001 (2)
C10	0.058 (4)	0.045 (3)	0.075 (3)	0.005 (2)	0.016 (3)	−0.003 (2)
C11	0.052 (3)	0.051 (3)	0.059 (2)	−0.002 (3)	0.021 (2)	0.001 (3)
C12	0.050 (3)	0.048 (3)	0.046 (2)	0.000 (3)	0.018 (2)	−0.002 (2)

### Geometric parameters (Å, °)

**Table:**

O1—C12	1.206 (4)	C3—C4	1.392 (4)
O2—C11	1.210 (3)	C3—H3	0.9300
O3—N3	1.244 (3)	C4—C5	1.371 (4)
O4—N3	1.251 (3)	C4—H4	0.9300
O5—N3	1.256 (3)	C5—H5	0.9300
N1—C5	1.335 (4)	C6—C7	1.385 (4)
N1—C1	1.347 (4)	C7—C8	1.381 (4)
N1—H1	0.8600	C7—C11	1.488 (4)
N2—C10	1.326 (4)	C8—C9	1.387 (4)
N2—C6	1.350 (4)	C8—H8	0.9300
C1—C2	1.387 (4)	C9—C10	1.389 (4)
C1—C6	1.467 (4)	C9—H9	0.9300
C2—C3	1.377 (4)	C10—H10	0.9300
C2—C12	1.492 (4)	C11—C12	1.559 (4)
			
C5—N1—C1	122.7 (3)	N2—C6—C7	124.1 (4)
C5—N1—H1	118.7	N2—C6—C1	114.7 (4)
C1—N1—H1	118.7	C7—C6—C1	121.2 (4)
C10—N2—C6	116.6 (3)	C8—C7—C6	118.5 (4)
O3—N3—O4	120.3 (3)	C8—C7—C11	120.9 (4)
O3—N3—O5	120.3 (3)	C6—C7—C11	120.5 (4)
O4—N3—O5	119.4 (3)	C7—C8—C9	117.8 (4)
N1—C1—C2	119.1 (4)	C7—C8—H8	121.1
N1—C1—C6	117.6 (4)	C9—C8—H8	121.1
C2—C1—C6	123.3 (4)	C8—C9—C10	119.8 (4)
C3—C2—C1	119.5 (4)	C8—C9—H9	120.1
C3—C2—C12	121.0 (4)	C10—C9—H9	120.1
C1—C2—C12	119.5 (4)	N2—C10—C9	123.1 (4)
C2—C3—C4	119.4 (4)	N2—C10—H10	118.4
C2—C3—H3	120.3	C9—C10—H10	118.4
C4—C3—H3	120.3	O2—C11—C7	123.4 (5)
C5—C4—C3	119.6 (4)	O2—C11—C12	118.6 (4)
C5—C4—H4	120.2	C7—C11—C12	118.0 (4)
C3—C4—H4	120.2	O1—C12—C2	122.5 (4)
N1—C5—C4	119.8 (4)	O1—C12—C11	120.0 (4)
N1—C5—H5	120.1	C2—C12—C11	117.5 (4)
C4—C5—H5	120.1		
			
C5—N1—C1—C2	0.5 (5)	N2—C6—C7—C11	−179.1 (3)
C5—N1—C1—C6	−178.2 (3)	C1—C6—C7—C11	1.2 (5)
N1—C1—C2—C3	0.0 (5)	C6—C7—C8—C9	0.7 (5)
C6—C1—C2—C3	178.6 (3)	C11—C7—C8—C9	179.5 (3)
N1—C1—C2—C12	−178.9 (3)	C7—C8—C9—C10	−1.0 (5)
C6—C1—C2—C12	−0.3 (5)	C6—N2—C10—C9	−0.5 (5)
C1—C2—C3—C4	−0.1 (5)	C8—C9—C10—N2	1.0 (6)
C12—C2—C3—C4	178.7 (3)	C8—C7—C11—O2	−0.4 (5)
C2—C3—C4—C5	−0.2 (5)	C6—C7—C11—O2	178.4 (3)
C1—N1—C5—C4	−0.8 (5)	C8—C7—C11—C12	178.9 (3)
C3—C4—C5—N1	0.6 (6)	C6—C7—C11—C12	−2.3 (5)
C10—N2—C6—C7	0.2 (5)	C3—C2—C12—O1	−0.9 (5)
C10—N2—C6—C1	179.8 (3)	C1—C2—C12—O1	178.0 (4)
N1—C1—C6—N2	−0.9 (5)	C3—C2—C12—C11	−179.6 (3)
C2—C1—C6—N2	−179.5 (3)	C1—C2—C12—C11	−0.8 (4)
N1—C1—C6—C7	178.7 (3)	O2—C11—C12—O1	2.5 (6)
C2—C1—C6—C7	0.1 (5)	C7—C11—C12—O1	−176.8 (3)
N2—C6—C7—C8	−0.3 (5)	O2—C11—C12—C2	−178.6 (3)
C1—C6—C7—C8	−179.9 (3)	C7—C11—C12—C2	2.1 (5)

### Hydrogen-bond geometry (Å, °)

**Table:**

*D*—H···*A*	*D*—H	H···*A*	*D*···*A*	*D*—H···*A*
N1—H1···O5	0.86	1.95	2.703 (4)	145
C8—H8···O5^i^	0.93	2.48	3.396 (4)	167
C9—H9···O4^i^	0.93	2.71	3.359 (5)	128
C4—H4···O4^ii^	0.93	2.61	3.323 (4)	134
C3—H3···O3^ii^	0.93	2.40	3.209 (4)	146
C10—H10···O1^iii^	0.93	2.47	3.318 (5)	151
C5—H5···O2^iv^	0.93	2.37	3.266 (5)	162

Symmetry codes: (i) *x*+1/2, *y*+1/2, *z*; (ii) *x*+1/2, *y*−1/2, *z*; (iii) *x*−1/2, *y*+1/2, *z*; (iv) *x*−1/2, *y*−1/2, *z*.
